# Proteome-wide alterations on adipose tissue from obese patients as age-, diabetes- and gender-specific hallmarks

**DOI:** 10.1038/srep25756

**Published:** 2016-05-10

**Authors:** María Gómez-Serrano, Emilio Camafeita, Eva García-Santos, Juan A. López, Miguel A. Rubio, Andrés Sánchez-Pernaute, Antonio Torres, Jesús Vázquez, Belén Peral

**Affiliations:** 1 Instituto de Investigaciones Biomédicas, Alberto Sols, (IIBM); Consejo Superior de Investigaciones Científicas & Universidad Autónoma de Madrid (CSIC-UAM), Madrid, 28029, Spain; 2 Laboratory of Cardiovascular Proteomics, Centro Nacional de Investigaciones Cardiovasculares (CNIC), Madrid, 28029, Spain; 3 Department of Endocrinology, Hospital Clínico San Carlos (IDISSC), Facultad de Medicina, Universidad Complutense, Madrid, 28040, Spain; 4 Department of Surgery, Hospital Clínico San Carlos (IDISSC), Facultad de Medicina, Universidad Complutense, Madrid, 28040, Spain; 5 CIBER de Fisiopatología de la Obesidad y Nutrición (CIBEROBN), Instituto de Salud Carlos III (ISCIII), Spain

## Abstract

Obesity is a main global health issue and an outstanding cause of morbidity and mortality predisposing to type 2 diabetes (T2DM) and cardiovascular diseases. Huge research efforts focused on gene expression, cellular signalling and metabolism in obesity have improved our understanding of these disorders; nevertheless, to bridge the gap between the regulation of gene expression and changes in signalling/metabolism, protein levels must be assessed. We have extensively analysed visceral adipose tissue from age-, T2DM- and gender-matched obese patients using high-throughput proteomics and systems biology methods to identify new biomarkers for the onset of T2DM in obesity, as well as to gain insight into the influence of aging and gender in these disorders. About 250 proteins showed significant abundance differences in the age, T2DM and gender comparisons. In diabetic patients, remarkable gender-specific hallmarks were discovered regarding redox status, immune response and adipose tissue accumulation. Both aging and T2DM processes were associated with mitochondrial remodelling, albeit through well-differentiated proteome changes. Systems biology analysis highlighted mitochondrial proteins that could play a key role in the age-dependent pathophysiology of T2DM. Our findings could serve as a framework for future research in Translational Medicine directed at improving the quality of life of obese patients.

Obesity, which is defined as an enlargement of the body fat due to an imbalance between energy uptake and expenditure, has reached epidemic proportions in the last decades and is now established as the most prevalent metabolic disease worldwide, affecting not only adults, but also children and adolescents^1^. White adipose tissue, a critical regulator of adiposity, is recognized not only as the main site of energy storage, but also as a complex, essential, and highly active metabolic and endocrine organ^2^. In humans, the adipose tissue is dispersed throughout the whole body with major intra-abdominal depots around the *omentum*, intestines, and perirenal areas as well as subcutaneous depots in the buttocks, thighs, and abdomen. These two main depots, the subcutaneous (SAT) and the visceral adipose tissue (VAT), exhibit differentiated structural, functional and biochemical properties^3^. Increased VAT, rather than the amount of SAT, has been found to be a major correlate of a cluster of diabetogenic, atherogenic, prothrombotic and proinflammatory metabolic abnormalities. Moreover, VAT accumulation has been linked to ectopic fat deposition in other body sites, such as the muscle, the liver and the heart^4^. Based on these evidences, visceral obesity has been established as a serious and complex phenotype largely connected with the development of several co-morbidities such as hypertension, dyslipidaemia, type 2 diabetes (T2DM) and cardiovascular diseases (CVD)^5^. Since T2DM emerges frequently in obese patients, its incidence is also increasing at an alarming rate worldwide^6^. The clinical characteristics of T2DM are usually shown several years after the onset of insulin resistance (IR), where the individual is usually asymptomatic^7^. Thus, early detection of T2DM, or preferably avoidance of disease onset, would be of tremendous benefit for human health.

Our group has been contributing to unveiling the human adipose tissue proteome for the last 10 years. We have previously linked obesity to attenuated VAT metabolism, marked down-regulation of mitochondrial functions and noticeable alteration of structural proteins, the latter accounting for the morphological changes observed in obese adipocytes^8^. While transcriptomic studies on human obesity are well-documented^9^, less attention has been paid to the proteome of obese adipose tissue. Nevertheless significant proteomic studies in obesity regarding mature adipocytes^10^,^11^, including metabolic models integrating transcriptomic and proteomic data^12^, have been addressed. Most of the studies have largely relied on a variety of experimental models of obesity and cultured adipocytes, and most of them have resorted to electrophoretic protein separation, which has very limited proteome coverage and results in a poor representation of low-abundant or very hydrophobic proteins as well as those with extreme pI and molecular weight^13^,^14^. On the other hand, current high-throughput proteomic techniques enable reliable detection and quantitation of thousands of proteins in complex proteomes without previous electrophoretic separation based on in-solution, in-gel or on-filter digestion of whole proteomes followed by peptide analysis by liquid chromatography coupled to mass spectrometry (LC-MS)^15^. In spite of their enormous potential, very few proteomic studies on obesity and its co-morbidities have resorted to these innovative approaches.

Here, we compare for the first time VAT from obese subjects to investigate the influence of T2DM in protein expression based on a high-throughput proteomics approach encompassing peptide labelling with isobaric tags for relative and absolute quantitation (iTRAQ) followed by LC-MS analysis. This multiplexed approach provides a wide dynamic range and proteome coverage, allowing the simultaneous quantitation of up to eight complex proteomes in the same experiment, which offers an invaluable advantage for the analysis of limited sample amounts^16^. Moreover, we have used a most rigorous statistical model recently developed *ad-hoc* for the treatment of quantitative proteomics data, the weighted spectrum, peptide and protein (WSPP) model^17^, which has been validated in a variety of proteomics experiments^18^,^19^,^20^. Using these state-of-the art proteomics methods we have quantitatively assessed not only the differences in VAT protein composition between diabetic and non-diabetic obese patients, but also whether gender accounts for significant dissimilarities in T2DM subjects, as wells as the influence of aging in obese adipose tissue. Given that aging is known to be a risk factor for obesity and T2DM^21^, and gender differences in adipose tissue distribution have been largely established^22^, the evaluation of the impact of aging and sexual dimorphism on the obese VAT proteome is of outmost interest.

The detailed depiction of the VAT proteome achieved, actually the most comprehensive to date, has unveiled a global down-regulation of mitochondrial proteins related to both T2DM development and aging progression, while, interestingly, the corresponding protein profiles in the two comparisons turned out to be essentially dissimilar. While the proteome alterations associated with aging were accounted for mainly by a dysfunctional electron transport chain (organelle level), the differences associated with T2DM involved a larger repertoire of mitochondrial functions, most likely resembling a decrease in the number of mitochondria (cellular level). Moreover, a distinctive gender-specific phenotype was discovered in T2DM patients, showing for the first time that after T2DM onset women suffer from a more dysfunctional adipose tissue as compared to men due to increased pro-inflammatory state and decreased VAT adipocyte hyperplasia. To gain an integrated comprehensive view of the VAT proteome dynamics systems biology analyses based on artificial intelligence and pattern recognition techniques^23^ were also performed, highlighting four mitochondrial proteins, related for the first time with human obesity, that could play a key role in the age-dependent pathophysiology of T2DM.

## Results

### The VAT proteome in obesity. Alterations as a function of age, T2DM and gender

**Figure 1:**
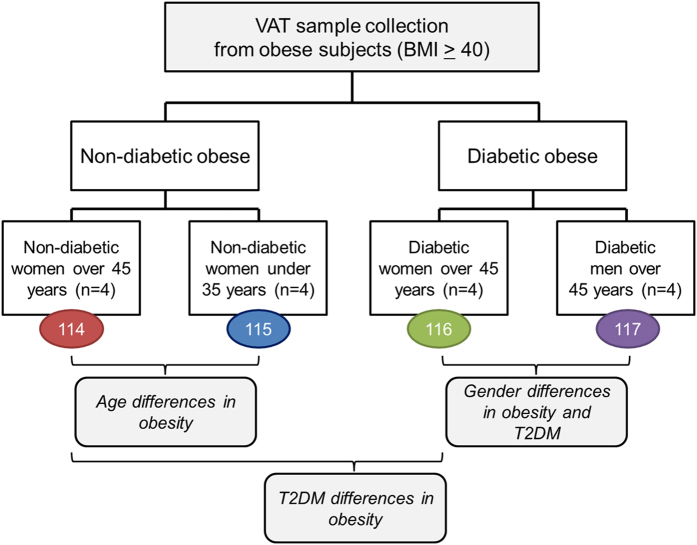
VAT samples and comparative proteomic studies. Two main groups of patients were considered: patients with T2DM (diabetic obese) and patients without T2DM (non-diabetic obese). Four groups (n = 4 each) were constituted as follows: non-diabetic obese women over 45 years, non-diabetic obese women under 35 years, diabetic obese women over 45 years and diabetic obese men over 45 years. Proteins were extracted from individual VAT samples, pooled in their corresponding group and digested. The peptide pools were tagged with iTRAQ labels (indicated with different colours) and mixed. The multiplexing capacity of iTRAQ technology allowed three differential expression studies.

For proteome profiling, VAT samples were collected from a subgroup of sixteen obese patients (Body Mass Index, BMI ≥ 40 kg/m^2^) with highly homogenous clinical features selected out of 50 subjects initially enrolled who underwent bariatric surgery. Two main groups were enclosed: patients with T2DM (diabetic obese, n = 8) and patients without T2DM or other obesity-associated co-morbidity (non-diabetic obese, n = 8). Each of these groups was split into two subgroups based on gender (diabetic women, n = 4; diabetic men, n = 4) and age (non-diabetic women over 45 years, n = 4; non-diabetic women under 35 years, n = 4) (Fig. 1). Patient clinical aspects are summarized in Supplementary Table S1. In view of the increased risk of suffering T2DM with age, together with the sexual dimorphism reported in obesity, we have compared age- and gender-matched groups in a single experiment.

At 1% peptide False Discovery Rate (FDR) 14,118 unique peptides corresponding to 2,525 unique proteins and codified by 2,329 human genes were identified (Supplementary Table S2A). Analysis of the major Gene Ontology Cellular Component terms (GOCCs) from these proteins revealed that the main organelles were represented, composed essentially by cytoplasmic (22%), plasma membrane (13%), nuclear (13%) and mitochondrial proteins (7%), with contribution also from the endoplasmic reticulum (4%) and the Golgi apparatus (2%) (Supplementary Fig. S1A). To assess the biological relevance of the proteins identified, enrichment analyses using DAVID software^24^ were performed. The GO Biological Process terms (GOBPs) found significantly enriched in the VAT proteome comprised well known adipose tissue functions such as *Generation of precursor metabolites and energy* or *Glucose and fatty acid metabolic process*, and obesity-related events such as *Response to oxidative stress* or *Response to unfolded protein* (Supplementary Fig. S1B and Table S2B), in agreement with previous studies on human VAT proteome^8^,^25^. Major mitochondrial pathways were also enriched.

**Figure 2:**
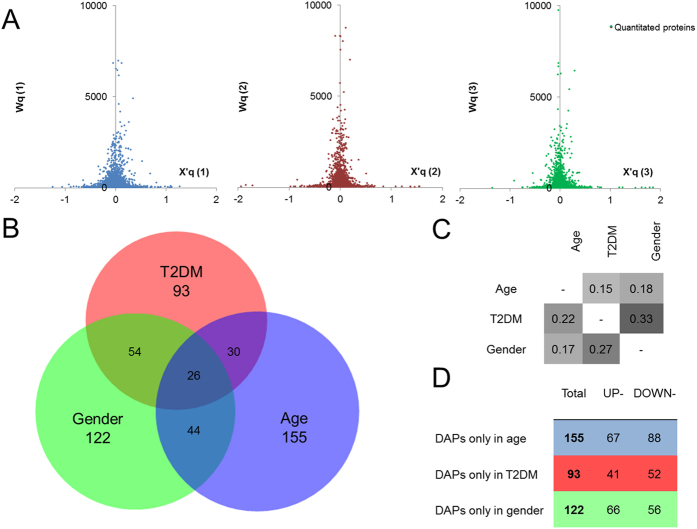
Protein abundance changes in VAT from obese patients. **(A)** Dot-plots of protein mean-corrected log_2_ ratios, X′q, *vs*. their corresponding statistical weight, Wq, showing normal distribution of data in each statistical comparison: age (1), T2DM (2) and gender (3). **(B)** Area-proportional Venn’s diagram showing the number of DAPs in the three statistical comparisons obtained with BioVenn software^72^. **(C)** Relative overlapping of DAPs among comparisons. **(D)** Number of total, up- and down-regulated DAPs specific to each comparison. For further information regarding protein changes and statistical values see also Supplementary Table S3.

The quantitative proteomics data in this study were analysed using the WSPP statistical model^17^. Plasma proteins and epidermal cytokeratins were excluded from the analysis to ensure a normal distribution of the dataset (Fig. 2A), which finally contained 12,824 unique peptides corresponding to 2,371 proteins codified by 2,245 different human genes (Supplementary Table S3). The model defines a standardized variable at the protein level, Zq, as the mean-corrected log_2_ ratio expressed in units of standard deviation. In this study, proteins with |Zq| ≥ 2 (corresponding to *p* < 0.05) were considered as differentially abundant proteins (DAPs) in each comparison.

**Figure 3:**
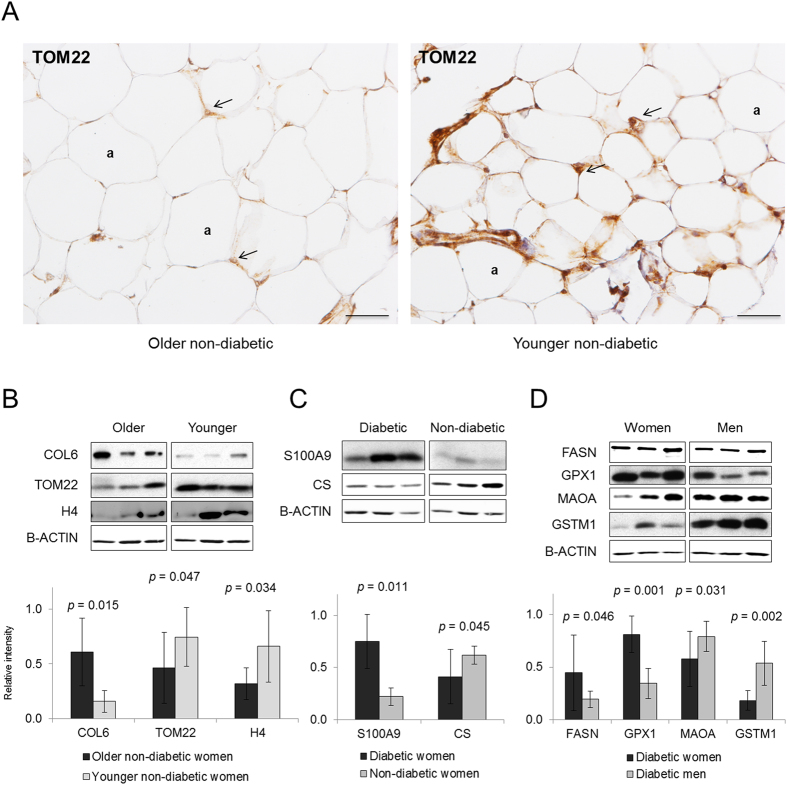
Western blot and immunohistochemistry analyses for DAP validation. **(A)** IHC detection of TOM22 in VAT samples from 4 older and 4 younger non-diabetic patients showing over-expression of TOM22 staining (in brown) in younger adipose cells, mainly surrounding the nuclei where the mitochondrial are located (arrows). Magnification x200 under Nikon Eclipse 90i microscope. a, adipocyte. Scale bars, 50 μm. **(B–D)** Representative WB analyses of selected DAPs using an additional set of 32 VAT samples (8 per cohort) in age, T2DM and gender comparisons. The results were normalized for B-actin density. Underneath, the values for relative intensity obtained after densitometry of the bands are means ± SD. Statistical significance was set at *p* < 0.05.

A total of 255 DAPs were found when comparing older (mean age 50 years) and younger women (mean age 32 years), while 203 proteins showed different abundance between diabetic and non-diabetic women and the level of 246 proteins revealed different in women as compared to men. Interestingly, 15–33% of the DAPs were common among the three comparisons (Fig. 2B,C). In particular, the T2DM and gender comparisons showed the greatest DAPs overlap, 33%, suggesting a high impact of gender on the diabetic phenotype. On the other hand, 100–150 DAPs were found to be specific in each comparison (Fig. 2D). These proteins were separately subjected to category enrichment analyses, where categories with *p* < 0.05 were considered as significantly enriched (Supplementary Tables S4, 5, 6). In the age comparison, *extracellular matrix* related terms were found over-represented with up-regulated proteins, whilst *translation*, *transcription* and *mitochondrion* related categories were found over-represented with down-regulated proteins (Table 1, Supplementary Table S4). These results revealed alterations of translation and transcription processes together with disturbances in the extracellular matrix and the mitochondrion with aging. To validate the pattern of protein abundance changes collagen VI (COL6), mitochondrial import receptor subunit TOM22 homolog (TOM22) and histone H4 proteins were analysed using Western Blot (WB) and immunohistochemistry (IHC) methods in an independent set of 16 VAT samples corresponding to older and younger obese patients. Results showed significant changes consistent with the above-described quantitative proteomics results (Fig. 3A,B).

In diabetic compared to non-diabetic patients, the categories found significantly more abundant were *Calcium binding, Immune response* and *Inflammation* with up-regulated proteins, while categories related to mitochondrial structure and function such as *Generation of precursor metabolites and energy, ATP synthesis* and *Pyruvate metabolism* were over-represented in the group of down-regulated proteins (Table 1, Supplementary Table S5). The up-regulation of S100 calcium-binding protein A9, S100A9, and the down-regulation of citrate synthase, CS, in diabetic compared to non-diabetic patients were validated by WB in an independent set of 16 diabetic and non-diabetic VAT samples (Fig. 3C).

The functional categories revealed differential between women and men add adipose tissue to the broad spectrum of physiological differences described between genders. Women showed over-expression of *Extracellular region* and *Immune response*-related terms, and men comprised up-regulated proteins mainly related to *Oxidation reduction* and *Glutathione transferase activity* (Table 1, Supplementary Table S6). WB analyses were performed in an additional set of 16 obese diabetic women and men for 4 proteins selected from the DAPs pertaining to two up-regulated and two down-regulated enriched categories in the gender comparison: fatty acid synthase (FASN), glutathione peroxidase 1 (GPX1), amine oxidase [flavin-containing] A (MAOA), and glutathione S-transferase Mu 1 (GSTM1). Results confirmed significant changes consistent with the above-described quantitative proteomics results (Fig. 3D).

### Impact of age, T2DM and gender on protein function dynamics in obesity

**Figure 4:**
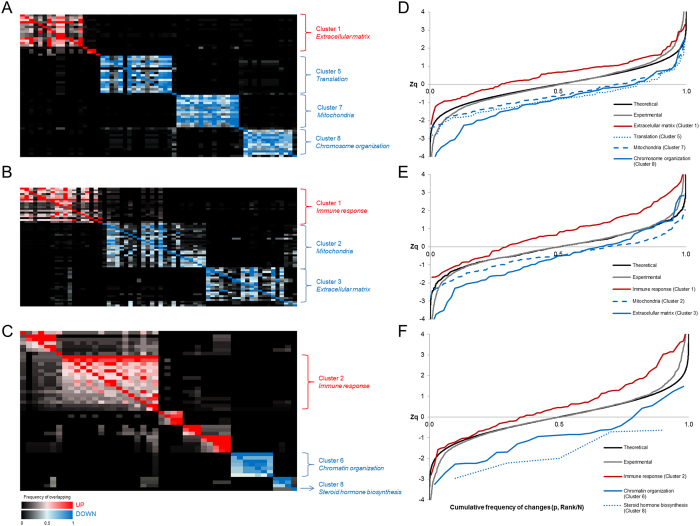
Protein function dynamics in obese patients. **(A–C)** Clustering of the functional categories altered in the age (**A**), T2DM (**B**) and gender (**C**) comparisons. A colour scale was used to represent up-regulated categories in red and down-regulated in blue. A detailed version of clustered categories is displayed in Supplementary Tables S7, S8, S9, respectively. **(D–F)** Alteration of representative cluster categories in the age (**D**), T2DM (**E**) and gender (**F**) comparisons. The cumulative frequency of changes (Zq) for the least redundant category in each of the clusters highlighted is represented together with a theoretical curve showing a normal distribution of data and the experimental curve representing Zq for the whole set of proteins quantified.

Most proteomic studies are focused on significant protein changes. This simplification has two serious drawbacks: i) its result strongly depends on the arbitrary threshold set for assessing DAPs; and ii) the vast majority of valuable quantitative information is overlooked. In contrast, the normal distribution of protein quantifications predicted by the WSPP statistical model under the null hypothesis is especially suitable for conducting ontological analyses of complete datasets without arbitrary thresholds. Thus, a functional category is considered up- or down-regulated at 5% FDR when the changes of its protein components fail to follow a normal distribution and show significantly up- or down-regulated, respectively (Supplementary Tables S7, S8, S9). Given the hierarchical design of functional annotations (*i.e*. the occurrence of parent and child GO categories), these often share protein components, which require clustering analyses based on the frequency of overlapping terms (Fig. 4A–C).

In the age comparison, a total of 62 categories were found regulated which generated eight category clusters (Fig. 4A, Supplementary Table S7). One of the largest clusters comprises categories related with *extracellular matrix* proteins (Cluster 1), where the majority of protein components were up-regulated in older *vs*. younger women. The other three large clusters including down-regulated proteins are related to *translation* (Cluster 5), *mitochondria* (Cluster 7) and *chromosome organization* processes (Cluster 8), in agreement with the above-described enrichment analysis. To illustrate the high degree of coordination of protein changes, the cumulative frequency distribution of changes, (Zq), was plotted for the least redundant categories in the cluster (Fig. 4D). Results indicate that the aforementioned protein function modulation highlighted by the reduced set of DAPs is substantiated by proteome-wide functional analysis.

In the T2DM assessment, 64 categories were found altered, leading to five category clusters (Fig. 4B, Supplementary Table S8). A cluster composed of up-regulated proteins in the diabetic *vs*. non-diabetic women related to the *immune response* can be easily recognized (Cluster 1), together with two additional large clusters including down-regulated proteins related to *mitochondria* (Cluster 2) and *extracellular matrix* (Cluster 3). In agreement with the enrichment analyses, the *mitochondria* cluster comprises not only central energy-related categories like *Oxidative phosphorylation*, but also characteristic mitochondrial metabolic pathways like *TCA cycle* and *Valine, leucine and isoleucine degradation*. Strikingly, proteome-wide changes related to the adaptation of mitochondrial metabolism take place to a similar magnitude in aging and T2DM, as can be observed in the relative shift patterns of the *mitochondria* cluster in Fig. 4D,E, respectively. However, the number of down-regulated proteins related to mitochondrial structure and function is higher in the T2DM than in the age comparison (Supplementary Tables S7 and S8). Additionally, network analysis of mitochondrial down-regulated proteins in the age and T2DM comparisons also revealed a larger repertoire of down-regulated functions in T2DM (Supplementary Fig. S2).

**Figure 5:**
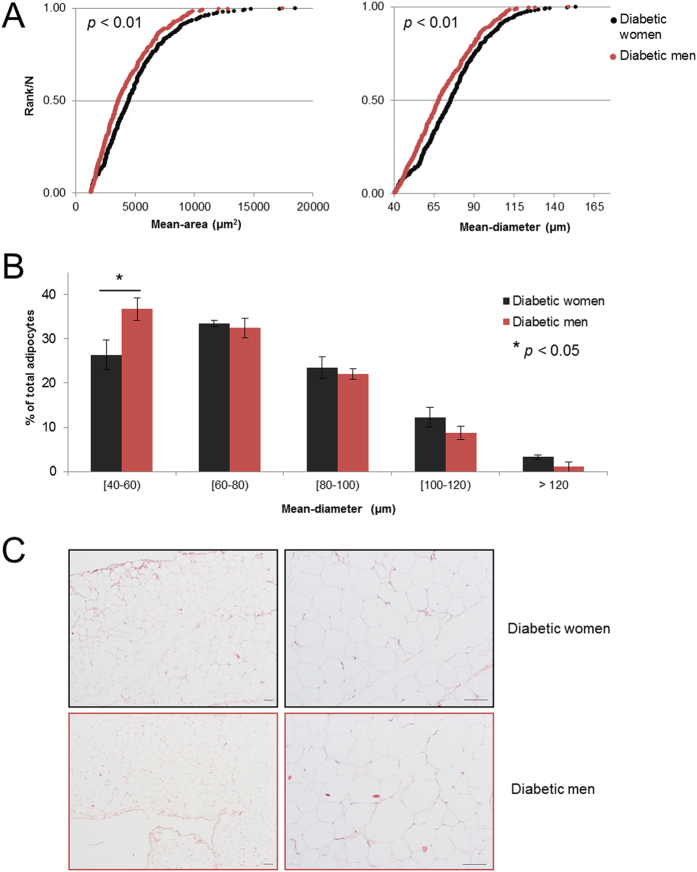
Evaluation of adipose tissue cellularity based on histological section analyses. Haematoxylin-eosin stained images from obese diabetic women (n = 4) and obese diabetic men (n = 4) were analysed using *Adiposoft* software. A total of 72 high-resolution images were randomly acquired for the analyses. Minimal and maximum threshold for automated measurement of adipocyte diameter was set at 40 μm and 175 μm, respectively. **(A)** Normal distribution of mean-area (μm^2^) and mean-equivalent diameter (μm) in diabetic women (black series, n = 327) and men (red series, n = 349) adipocytes from a representative experiment. **(B)** % of total adipocytes according to different mean-equivalent diameter ranges (in μm). Bars represent the average of the relative percent of adipocytes in each range ± SD from each group among three independent experiments. Statistical significance between diabetic women and men was set at *p* < 0.05. **(C)** Representative haematoxylin-eosin fields at x40 (left panel) and x100 (right panel) magnification used for adipocyte size estimation in both diabetic women and men. Scale bars, 100 μm.

The gender comparison yielded 46 regulated categories grouped into eight clusters (Fig. 4C, Supplementary Table S9). The largest cluster comprises categories related to *immune response* (Cluster 2) with up-regulated proteins in women compared to men (Fig. 4F). Of note, the up-regulation of proteins involved in *steroid hormone biosynthesis* (Cluster 8) in men highlight the androgenic phenotype at the adipose tissue level. Altogether, enrichment and clustering analyses suggested a gender-dependent plasticity of adipose tissue. In addition, analyses of adipose tissue cellularity in histological sections showed significant changes between women and men (Fig. 5). Female adipocytes were significantly larger than those from males as revealed by increased mean-area and mean-equivalent diameter (*p* < 0.01), confirming cellular hypertrophy in women (Fig. 5A). Meanwhile, the fraction of smallest adipocytes (40–60 μm diameter) was significantly higher in males (Fig. 5B), suggesting a more hyperplasic phenotype in men as compared to women (*p* < 0.05).

### Systems biology approach to the prediction of protein function alterations in T2DM and aging

**Figure 6:**
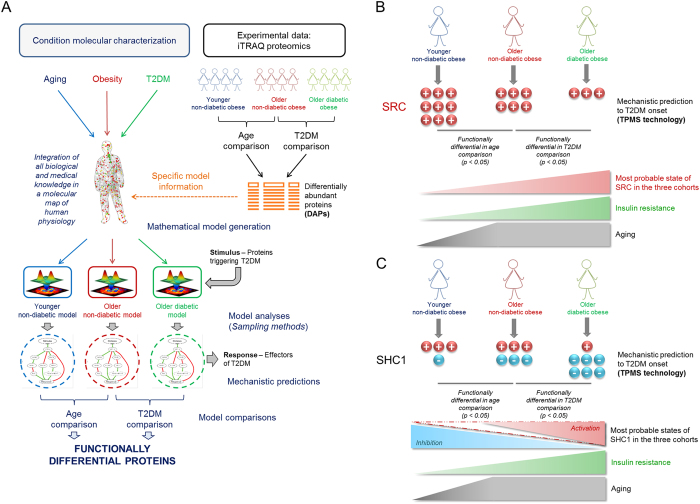
Systems biology approach to the prediction of protein function alterations in T2DM and aging. **(A)** Computational modelling according to TPMS technology. The DAPs from the iTRAQ-based proteomic study were analysed through computational modelling of 3 cohorts: non-diabetic obese women under 35 years, non-diabetic obese women over 45 years and diabetic obese women over 45 years. Three mathematical models were generated and each of them was challenged with the stimulus (the most relevant proteins triggering T2DM) and the response (T2DM effectors). The most probable molecular pathways leading from the stimulus to the response through the biological network were traced, revealing the most probable molecular mechanisms to develop T2DM in each of the three cohorts. By comparing the mathematical models, a set of functionally differential proteins was obtained (*p* value < 0.05). A detailed description of TPMS technology can be found in Supplemental Experimental Procedures. **(B,C)** Mechanistic predictions related to the onset of T2DM and hypothetical interpretation of data. (**B**) SRC is more activated in both non-diabetic (T2DM comparison) and younger women (age comparison). (**C**) SHC1 inhibition was predicted as over-represented in older (age comparison) and diabetic patients (T2DM comparison). Conversely, SHC1 activation was also over-represented in older non-diabetic women (T2DM comparison). We acknowledged Anaxomics Biotech. for panel (**A**) drawings.

A remarkably different pattern of mitochondrial dysfunction has been evidenced in the above-described T2DM and age comparisons. To further explore this result, the quantitative proteomics data obtained from three cohorts (younger non-diabetic women, older non-diabetic women and older diabetic women) were subject to computational modelling based on artificial intelligence and pattern recognition techniques. The *Therapeutic Performance Mapping System* (TPMS, Anaxomics Biotech.)^26^,^27^,^28^ was used to generate a mathematical model for each cohort based on the DAPs identified in each comparison (Fig. 6A). Each model was challenged with a stimulus and a response, providing for each cohort a population of solutions, *i.e*. sets of the protein functional states explaining the network perturbation from the stimulus to the response. In our case, the network was stimulated with the functional modulation of the most relevant proteins triggering T2DM while the response was set as the disease-causing functional modulation of the whole set of proteins characterized as involved in T2DM. The proteins found more functionally affected in one particular direction (activation or inhibition) in the model solutions of one cohort compared to the other (older *vs*. younger and diabetic *vs*. non diabetic) were identified as differentially modulated proteins in that cohort.

TPMS discovered 40 functionally differential proteins (*p* < 0.05) with altered activation states between the models compared, four of which were mitochondrial proteins: proto-oncogene tyrosine-protein kinase Src (SRC), SHC-transforming protein 1 (SHC1), TNF receptor-associated factor 3 (TRAF3) in the age assessment; and SRC, SHC1 and mitogen-activated protein kinase 10 (MAPK10) in the T2DM comparison (Supplementary Table S10).

## Discussion

Obesity is a highly heterogeneous condition in which body fat distribution, especially VAT accumulation, termed visceral obesity, has been found to correlate with metabolic diseases such as T2DM and CVD^5^. While men have a more harmful obese phenotype as compared to women, aging influences fat deposition with significant increase in total fat mass, re-distribution of body adipose tissue with enlarged VAT and decline in SAT, as well as ectopic fat deposition. Our previous studies have linked obesity to an attenuated metabolism of adipose tissue and a marked down-regulation of mitochondrial functions^8^, while other works have also correlated obesity and related co-morbidities with mitochondrial dysfunction of the adipose tissue^29^,^30^. Moreover, mitochondrial dysfunction has long been considered a major contributor to aging and age-related diseases involving both reactive oxygen species (ROS) production and other aspects of mitochondrial physiology such as mitochondrial biogenesis and turnover, energy sensing, apoptosis, senescence or calcium dynamics^31^.

To deepen the knowledge of T2DM development in obesity and the influence of both aging and gender in the human adipose tissue, we have resorted to a non-hypothesis-driven strategy that combines a high-throughput proteomics platform, namely iTRAQ labelling and LC-MS, and systems biology tools. Our experimental design has allowed not only the extremely robust assessment of protein abundance changes (for which adequate statistics are essential^16^), but also considerably lower sample amount requirements and LC-MS data acquisition time compared to other proteomics methods. To our knowledge, this is the first report that relies on these approaches to assess T2DM, aging and gender differences in adipose tissue from obese patients.

In older compared to younger patients, enrichment analysis revealed a significant down-regulation of terms related to protein synthesis, chromatin remodelling, mRNA processing, mRNA binding and nucleosome ribosomal proteins, while *translation* and *chromosome organization* were revealed as two of the most over-represented events of down-regulated categories. Changes in chromatin architecture, such as histone modifications^32^ or global heterochromatin loss and redistribution^33^, have been shown to display characteristic features of aging. Based on proteomic approaches, we have detected the same under-expressed processes in human adipose tissue in relation to aging.

The age-based comparison also unveiled the up-regulation of proteins and categories related to extracellular matrix organization, underlining *extracellular matrix* as the largest cluster of over-represented categories associated with aging. The adipose tissue is a plastic organ in which extensive remodelling, extracellular matrix expansion and up-regulation of its protein components, as previously reported for COL6^34^, take place in response to both aging and obesity, where inappropriately increased and rigid extracellular matrix hinders adipose tissue growth^35^. The *steroid hormone biosynthesis* cluster was also up-regulated in older compared to younger women suggesting a key role for adipose tissue as an extra-gonadal source of steroids over time, in agreement with previous work^36^.

Interestingly, down-regulated proteins involved in mitochondrial structure and function have been found in aging and T2DM assessments. While the relation between mitochondrial dysfunction and T2DM has been extensively reported^37^,^38^, its long-suspected association with aging remains to be clarified. As cells and organisms age, the efficacy of the respiratory chain tends to diminish, thus increasing electron leakage and reducing ATP generation^39^. Indeed, mitochondrial dysfunction has been described as one of the nine hallmarks of aging^40^. The down-regulation of DAPs related to mitochondrial transport and membrane organization in older *vs*. younger patients illustrates that mitochondrial cellular components and molecular trafficking alterations directly impact intra- and inter-organelle crosstalk, and therefore intra- and inter-cellular endocrine communication^41^. Our results also highlight a proteome-wide down-regulation of functional categories related to mitochondrial energy metabolism, suggesting a key role for dysfunctional oxidative phosphorylation complexes in older *vs*. younger subjects in agreement with previous studies^40^. Remarkably, the categories encountered under-represented in diabetic *vs*. non-diabetic patients highlight an adjustment of typical mitochondrial processes such as *TCA cycle* and *ATP synthesis coupled to proton transport* which were not found in the age-based comparison. To deepen this result, CS was found down-regulated in T2DM (Fig. 3C, Table 1), in agreement with previous studies^42^. Moreover, in the T2DM assessment our proteomics study based on human VAT has also unveiled calcium signalling alterations, which have been linked to the development of IR at the mitochondrion and endoplasmic reticulum levels^43^. In particular, S100A9 has revealed significantly over-expressed in diabetic compared to non-diabetic subjects (Fig. 3C, Table 1), as earlier reported^44^.

To our knowledge, this is the first time that a proteome-wide down-regulation of mitochondrial proteins is reported in aging and T2DM, as shown in the relative shift patterns of the *mitochondria* clusters in Fig. 4D,E. Of note, the age- and T2DM-based comparisons led to dissimilar categories related to down-regulated mitochondrial proteins (Table 1, see also Supplementary Tables S4 and S5) together with dissimilar under-expressed categories in the functional and network analyses (Supplementary Fig. S2 and Tables S7 and S8). In aging, mitochondrial dysfunction is accompanied by features related to adipose tissue remodelling, such as increased extracellular matrix or up-regulation of PPAR signalling mediators. In T2DM, however, a larger repertoire of mitochondrial functions has been identified, most likely resembling a reduced number of mitochondria as previously reported^37^,^45^, which also correlates with decreased extracellular components and down-regulated PPAR mediators. These differences are likely related to the prodromal *vs*. pathological state in non-diabetic *vs*. diabetic patients, respectively. Causative inferences for mitochondrial dysfunction are difficult to achieve given the cross-sectional design of this study; nevertheless, the mitochondrial alterations caused by aging could reflect the early stages of IR development, as this adjustment is also accompanied by other well-recognized changes like the extracellular matrix deposition. Longitudinal analyses in non-diabetic patients over time would help clarify these issues.

Well-supported gender differences in adipose tissue distribution and metabolic risk have been found. In men, the adipose tissue localizes mainly to the abdominal region, accounting for a larger risk of suffering metabolic disorders^46^. Women are generally characterized by less VAT and more SAT^47^, as well as by a lower risk of developing obesity-associated co-morbidities. However, female visceral adiposity increases over time, resulting from a shift towards a central body fat distribution that increases the risk of T2DM compared to men^48^. Although some evidences have suggested that variation between men and women would be predominantly sex hormone-dependent, other features such as immune response dissimilarities, genes located on sex chromosomes, or specific gender conducts could contribute to these differences^49^.

Proteomic analyses addressing gender differences in adipose tissue are scarce. To our knowledge, this is the first proteomic study in which gender differences are reported in VAT from T2DM patients. The large DAP overlapping (33%) found between T2DM and gender comparisons suggests a key role for gender in the diabetic phenotype. Thus, *immune response* up-regulated function is over-represented in both the T2DM and the gender comparisons (Fig. 4B,C). Despite that increased immune response and a pro-inflammatory state are associated with T2DM our results suggest a specific role for immune response in women.

Protein abundance changes also revealed a distinctive male and female phenotype regarding antioxidant response (Table 1). Thus, SOD1, SOD3 and several GST proteins were found up-regulated in men, whereas ROS-scavenging GPX1 and GPX3 were up-regulated in women. The differential expression of several proteins corresponding to *Oxidation reduction* and *Glutathione transferase activity* categories between women and men were revealed by the above-described proteomic procedures and validated by an orthogonal method (Fig. 3D). As far as we know, this is the first time that a gender-specific antioxidant protein pattern is described in T2DM patients. On the other hand, results indicate that once T2DM has been established, no gender differences in the mitochondrial proteome can be observed.

The adipose tissue contributes to the circulating sex hormones^36^. Low levels of testosterone are associated with IR in men, while increased androgen levels induce IR and T2DM in women^50^. This gender specificity is found in the polycystic ovary syndrome, where female hyperandrogenism is related to IR and T2DM^51^. Our results involving the up-regulation of proteins related to steroid hormone biosynthesis in men (mainly characterized by C1, C2 and C3 members of aldo-keto reductase family 1, AKR1), as previously reported^52^, illustrate the androgenic phenotype in VAT, providing novel insights into adipose tissue steroid-converting enzymes. This androgenic phenotype was associated with the up-regulation of proteins involving chromatin organization which, in turn, could underlie improved adipocyte hyperplasic response in males as compared to females. Noteworthy, our data have also shown that FASN is significantly over-expressed in women compared to men (Fig. 3D, Table 1), supporting that increased VAT in women correlate with hypertrophic adipocytes. In fact, FASN, involved in lipid biosynthesis, has been long associated to hypertrophic visceral fat accumulation^53^, strengthening our results. In addition, histological analysis highlighted a significant enlargement of adipocyte size (adipocyte hypertrophy, Fig. 5A) in women, while men show more numerous and smaller adipocytes (adipocyte hyperplasia, Fig. 5B). Consequently, VAT in diabetic obese women as compared to men encloses a higher number of enlarged adipocytes which would exceed their vascular supply, leading to local hypoxia, fibrosis, adipocyte dysfunction, and cell death^54^.

Taken together, our data suggest a worsening of the obese phenotype in women once T2DM emerges due to increased pro-inflammatory state and decreased VAT adipocyte hyperplasia compared to men. Accordingly, previous studies have shown that diabetic women have poorer glycaemic control and are less likely to reach the goals for HbA1c than men^55^, despite that females demonstrate better adherence to diabetes care^56^. As far as we know this is the first time that a proteomic study reflects that after T2DM onset women suffer from a more dysfunctional adipose tissue as compared to men of similar age.

Additionally, we have resorted to systems biology approaches to gain insight into VAT functional changes, particularly into those related to mitochondrial regulation, revealed in this study as a common hallmark of aging and T2DM in obese patients. Four mitochondrial proteins were identified as differentially modulated in the age and T2DM assessments (Supplementary Table S10). Our data have shown that SRC is over-activated in both non-diabetic and younger women, therefore suggesting that SRC activation might play a key role in the age-dependent pathophysiology of T2DM. SRC is a redox sensitive enzyme^57^ whose activity is influenced by several regulators or by-products of oxidative phosphorylation related to the aging process. Accordingly, we hypothesize that with the onset of IR the activation of SRC is increased, since T2DM development requires higher levels of SRC activation in younger non-diabetic patients (more insulin sensitive) as compared to older non-diabetic ones (less insulin sensitive) (Fig. 6B). Interestingly, these evidences have been confirmed by studies showing that SRC activation promotes IR through Jun N-terminal kinase activation^58^.

Computational modelling showed that both the activated and inhibited functional states of SHC1 were found statistically differential among the groups of patients (Supplementary Table S10). Our data suggest that the modulation pattern of SHC1 with aging and IR onset requires greater loss of SHC1 inhibition together with increased SHC1 activation in diabetic compared to non-diabetic patients (Fig. 6C). SHC1 gene encodes three isoforms of 66, 52 and 46 kDa; while p46Shc and p52Shc isoforms are coupled to growth and survival signals, p66Shc mediates pro-apoptotic responses to oxidative stress^59^ being considered as a negative determinant of life span due to its effects on mTOR/S6K cascade promoting obesity and IR^60^. Our results suggest that more than one SHC1 isoform could be contributing to T2DM with a certain dependence of age. While SHC1 inhibition could be mostly related to p66Shc, SHC1 activation could be related to p46Shc isoform, accordingly to previous data based on mouse models studies^61^.

Our results suggest that inhibition of TRAF3, a negative regulator of inflammation^62^, would lead to the development of T2DM in non-diabetic obese patients. In agreement with this observation, TRAF3 has been recently suggested to undergo a functional switch toward pro-inflammatory modes in obesity^63^, hence coupling over-nutrition to metabolic inflammation, IR, and metabolic disease. Finally, the inhibition of MAPK10 (also named JNK3) in non-diabetic patients could be involved in the development of T2DM. MAPK10 plays a protective role in insulin-secreting cells against death caused by pro-inflammatory cytokines^64^. Since chronic inflammation is a well-recognized feature of obesity-related co-morbidities^65^, our results suggest that MAPK10 inhibition might be related to the impairment of analogous processes in VAT. Further characterization of these four differentially modulated proteins in VAT from a wide range of obese patients (diabetic, non-diabetic, older and younger) might contribute to clarify these fundamental issues.

The primary limitation of this study is the reduced number of patients finally enrolled after selecting those with highly homogeneous clinical features. It must be noted, however, that owing to the difficulties inherent to obtaining patient tissue samples, most clinical proteomics studies are based on relatively small patient cohorts (see for example^25^,^66^,^67^). In addition, the detrimental effect of the relatively limited number of samples is lessened by the application of a robust proteomic procedure and the validated WSPP statistical model. Unquestionably, our conclusions would have been reinforced by the inclusion of other patient groups such as younger obese women with T2DM; however, it must be taken into account that obese patients manifest T2DM several years after the onset of the IR early signs^7^. The enrolment of obese men without T2DM would have also strengthened our results; nevertheless, while female patients consult for obesity before they develop co-morbidities, male patients delay consultation until they experience metabolic and cardiovascular complications^68^. Likewise, although mitochondrial dysfunction has been highlighted as a central event in T2DM and aging, and therefore constituted the main focus in our systems biology analysis, proteomics has revealed a wide array of biomarkers with a potential role in obesity, T2DM and aging that warrant further investigation.

Our innovative combination of state-of-the art proteomics and systems biology approaches has provided much insight into the interplay among obesity, T2DM, aging and gender by revealing a striking proteome-wide down-regulation of mitochondrial functionality in both diabetic and older obese individuals, together with a well-defined gender-specific phenotype in obesity-related T2DM. These findings can contribute to the identification of therapeutic targets aimed at improving the quality of life of obese patients in the near future.

## Material and Methods

### Ethic statement

The study was conducted according to the recommendations of the Declaration of Helsinki and was approved by the Ethics Committees of Hospital Clínico San Carlos (Madrid, Spain). Signed informed consent was obtained from all subjects.

### Biological samples

VAT samples were collected from a subgroup of 16 obese patients (BMI ≥ 40 kg/m^2^) with highly homogenous clinical features selected out of 50 subjects initially enrolled. All the patients were of Caucasian origin and underwent bariatric surgery. The biopsies were obtained after an overnight fast, washed three times in PBS, partitioned into pieces, and immediately frozen in liquid nitrogen and stored at −80 °C until protein extraction. The surgeon aimed to obtain the samples at the beginning of the surgery and from the same anatomical location (*omentum)* in all subjects. All patients reported that their body weight had been stable for at least three months before the study. None of the non-diabetic subjects suffered T2DM or other obesity associated co-morbidity (hypertension, dyslipidemia, obstructive sleep apnea syndrome or CVD), and none of them was being treated. Inclusion criterion for the diabetic group was suffering from diabetes for at least two years. Diabetes was defined by fasting plasma glucose ≥7 mmol/L and HbA1c ≥ 6.5% (47.5 mmol/mol). All diabetic subjects were being treated with oral anti-diabetic drugs and in two cases with insulin in order to control the co-morbidities. The clinical characteristics of the patients enrolled are described in Supplementary Table S1. Exclusion criteria in this study are defined in Supplementary Information.

### Preparation of protein extracts and on-filter digestion

Proteins were extracted from VAT (about 100 mg) in radioimmunoprecipitation assay buffer (RIPA) (25 mM Tris-HCl pH 7.6, 150 mM NaCl, 1% NP-40, 1% sodium deoxycholate, 0.1% SDS) using the *Sample Grinding Kit* (GE Healthcare). Protein concentration was measured using *Pierce* BCA Protein Assay (Thermo Scientific) and *Direct Detect* (EMD Millipore). A protein pool was prepared with proteins from the four patients included in each group. One-hundred μg of proteins from each pool were digested on-filter (FASP, *Protein Digestion Kit*).

### iTRAQ labelling and MCX peptide fractionation

Proteomics procedures are scheduled in Supplementary Fig. S3 and fully described in Supplementary Information. The peptide pools were isobarically labelled (iTRAQ, Sigma-Aldrich), equally mixed and the peptide mixture was desalted onto Oasis HLB C18 cartridges (Waters). One-fifth of the tagged peptides were dried-down, and the remaining four-fifths were separated into 8 fractions using MCX Oasis cartridges (Waters). The peptide fractions were desalted using MicroSpin Colums C18 (The Next Group) and vacuum-dried.

### LC-MS analyses and protein identification

High-resolution analysis of iTRAQ-labeled peptides was carried out on an Easy nLC 1000 nano-HPLC apparatus (Thermo Scientific) coupled to a hybrid quadrupole-orbitrap mass spectrometer (Q Exactive, Thermo Scientific). A total of 7 MS data sets, 1 from the unfractionated material and 6 from the corresponding MCX fractions, were registered with 28 h total acquisition time. For peptide identification the MS/MS spectra were searched with the SEQUEST algorithm implemented in Proteome Discoverer 1.4.0.29 (Thermo Scientific).

### Statistical analyses

Descriptive results of continuous variables are expressed as mean ± standard deviation (SD). Statistical analyses were performed using the Statistical Package for Social Science software (v. 22, SPSS, Inc). One-way ANOVA (using Bonferroni’s *post hoc* test) was used to compare the anthropometrical and clinical data from the patients, and the statistical significance was set at *p* < 0.05. Peptide and protein abundance changes were assessed at 1% peptide FDR using the WSPP model^17^. This model provides a standardized variable, Zq, which is defined as the mean-corrected log_2_ ratio expressed in units of standard deviation at the protein level. The threshold for differential protein abundance was set at |Zq| ≥ 2 (corresponding to *p* < 0.05). In addition, *ca*. 2.3 · 10^6^ functional category terms with relevance to adipose tissue and obesity were retrieved from the DAVID database^24^ and used with the WSPP model to determine statistically significant changes at the protein function level as previously described^18^,^19^,^69^.

### Immunoblotting

Fat tissue was homogenized in RIPA buffer and protein extracts (10 μg) were loaded, resolved on SDS-PAGE and transferred to Hybond ECL nitrocellulose membranes as described^8^. Membranes were stained with 0.15% Ponceau red to ensure equal loading after transfer, and then blocked with 5% (w/v) BSA in TBS buffer with 0.1% Tween 20. The primary antibodies used were 1:2000 rabbit anti-COL6 and 1:10000 rabbit anti-total H4 (Abcam); 1:2000 mouse anti-FASN (BD Biosciences); 1:500 rabbit anti-TOM22, 1:5000 rabbit anti-CS and 1:1000 rabbit anti-GPX1 (Sigma-Aldrich); 1:1000 goat anti-S100A9; 1:2000 rabbit anti-MAOA, 1:250 rabbit anti-GSTM1 and 1:2000 goat anti-β-actin (Santa Cruz Biotechnology). Blots were incubated with the appropriate IgG-HRP-conjugated secondary antibodies. The immunoreactive bands were visualized with *ECL-plus reagent kit* (GE Healthcare). Blots were exposed for different times. Exposures in the linear range of signal were selected for densitometric evaluation. Optical densities of the immunoreactive bands were measured using *Image J analysis* software. In addition, infrared labelled secondary antibodies were used: donkey anti-goat IRDye 800 IgG (H + L), donkey anti-mouse IRDye 680RD IgG (H + L) and goat anti-rabbit IRDye 680CW IgG (H + L) (Li-Cor Biosciences). Here, the bound complex was detected using the Odyssey Infrared Imaging System (Li-Cor Biosciences). The images were analyzed using the *Odyssey Application Software* to obtain the integrated intensities. Statistical comparisons of the densitometry data were made with the independent t-test. Statistical significance was set at *p* < 0.05.

### Immunohistochemistry

Five-micron sections of formalin-fixed paraffin-embedded VAT were deparaffinized and rehydrated prior to antigen unmasking. Sections were blocked in normal serum and incubated overnight with 1:200 rabbit anti-TOM22 (Sigma-Aldrich). Secondary antibody staining was performed using the VECTASTAIN ABC kit and detected with diaminobenzidine (DAB) (both from Vector Laboratories). Sections were counterstained with haematoxylin prior to dehydration and cover-slip placement, and examined under a Nikon Eclipse 90i microscope. As a negative control, the procedure was performed in the absence of primary antibody.

### Analyses of histological sections for adipose tissue cellularity evaluation

Five-micron sections were stained with haematoxylin-eosin under conventional procedures. Histological sections were imaged at x40 and x100 magnification with a Nikon Eclipse 90i microscope in an automated fashion and analyzed using *Adiposoft* software^70^. Statistical comparisons between groups were made using independent t-test. Statistical significance was set at *p* < 0.05.

### Therapeutic Performance Mapping System (TPMS) analyses

Mathematical models that simulate human physiology *in silico*^26^,^27^,^71^ were generated by TPMS (Anaxomics Biotech.) in a four-step process: 1) Hand-curated collection of scientific knowledge relating biological processes to their molecular effectors; 2) Human biological network; 3) Mathematical model generation (including all linkable human proteins, and customized to fit the behaviour of different cohorts through introduction of quantitative proteomics results, *i.e*. DAPs); and 4) Extraction of biological conclusions by comparing protein activation states between models (older *vs*. younger and diabetic *vs*. non-diabetic women). Computational modelling through TPMS is fully described in Supplementary Information.

## Additional Information

**How to cite this article**: Gómez-Serrano, M. *et al*. Proteome-wide alterations on adipose tissue from obese patients as age-, diabetes- and gender-specific hallmarks. *Sci. Rep*. **6**, 25756; doi: 10.1038/srep25756 (2016).

## Supplementary Material

Supplementary Information

Supplementary Table S1

Supplementary Table S2

Supplementary Table S3

Supplementary Table S4

Supplementary Table S5

Supplementary Table S6

Supplementary Table S7

Supplementary Table S8

## Figures and Tables

**Table 1:** Representative enriched categories with some of their DAP components.

	*p* value	UniProt	Protein name	Symbol	Zq
Age comparison					
*Extracellular matrix* (GO:0031012)	0.00	P14780	Matrix metalloproteinase-9	MMP9	4.25
		Q12805	EGF-containing fibulin-like extracellular matrix protein 1	EFEMP1	3.54
		P55268	Laminin subunit beta-2	LAMB2	2.90
		P51888	Prolargin	PRELP	2.64
		P02462	Collagen alpha-1(IV) chain	COL4A1	2.45
		P12110	Collagen alpha-2(VI) chain	COL6A2	2.37
*Chromatin assembly or disassembly* (GO:0006333)	0.00	P16401	Histone H1.5	HIST1H1B	−2.16
		P62805	Histone H4	HIST1H4A	−2.69
		Q02539	Histone H1.1	HIST1H1A	−3.04
*Mitochondrial part* (GO:0044429)	0.04	Q8NC60	Nitric oxide-associated protein 1	NOA1	−2.20
		P06241	Tyrosine-protein kinase Fyn	FYN	−2.21
		P24310	Cytochrome c oxidase subunit 7A1, mitochondrial	COX7A1	−2.22
		Q9NS69	Mitochondrial import receptor subunit TOM22 homolog	TOMM22	−2.27
		P51970	NADH dehydrogenase [ubiquinone] 1 alpha subcomplex subunit 8	NDUFA8	−2.28
		P53007	Tricarboxylate transport protein, mitochondrial	SLC25A1	−2.42
T2DM comparison					
*Calcium ion binding* (GO:0005509)	0.00	P06702	Protein S100-A9	S100A9	3.61
		P07996	Thrombospondin-1	THBS1	3.25
		P05109	Protein S100-A8	S100A8	3.05
		P55083	Microfibril-associated glycoprotein 4	MFAP4	2.81
		P13796	Plastin-2	LCP1	2.06
		P06703	Protein S100-A6	S100A6	2.02
*Mitochondrion* (GO:0005739)	0.00	P99999	Cytochrome c	CYCS	−2.01
		O75964	ATP synthase subunit g, mitochondrial	ATP5L	−2.02
		P50213	Isocitrate dehydrogenase [NAD] subunit alpha, mitochondrial	IDH3A	−2.04
		O75947	ATP synthase subunit d, mitochondrial	ATP5H	−2.11
		Q5VTU8	ATP synthase subunit epsilon-like protein, mitochondrial	ATP5EP2	−2.12
		O75390	Citrate synthase, mitochondrial	CS	−2.27
		Q02252	Methylmalonate-semialdehyde dehydrogenase [acylating], mitochondrial	ALDH6A1	−2.27
		P05091	Aldehyde dehydrogenase, mitochondrial	ALDH2	−2.29
		Q5VT66	Mitochondrial amidoxime-reducing component 1	MARC1	−2.43
		P38117	Electron transfer flavoprotein subunit beta	ETFB	−2.44
Gender comparison					
*Inflammatory response* (GO:0006954)	0.00	P02763	Alpha-1-acid glycoprotein 1	ORM1	5.44
		P04003	C4b-binding protein alpha chain	C4BPA	3.67
		P05155	Plasma protease C1 inhibitor	SERPING1	3.15
		P10909	Clusterin	CLU	2.73
		P02751	Fibronectin	FN1	2.42
*Oxidation reduction* (GO:0055114)	0.01	P49327	Fatty acid synthase	FASN	5.59
		Q53TN4	Cytochrome b reductase 1	CYBRD1	4.35
		P00450	Ceruloplasmin	CP	4.21
		P07203	Glutathione peroxidase 1	GPX1	2.94
		O75891	Cytosolic 10-formyltetrahydrofolate dehydrogenase	ALDH1L1	2.69
		P22352	Glutathione peroxidase 3	GPX3	2.03
*Oxidation reduction* (GO:0055114)	0.00	P40939	Trifunctional enzyme subunit alpha, mitochondrial	HADHA	−2.03
		Q13162	Peroxiredoxin-4	PRDX4	−2.73
		P00441	Superoxide dismutase [Cu-Zn]	SOD1	−2.81
		P00352	Retinal dehydrogenase 1	ALDH1A1	−3.08
		P07195	L-lactate dehydrogenase B chain	LDHB	−3.09
		P21397	Amine oxidase [flavin-containing] A	MAOA	−3.16
		P08294	Extracellular superoxide dismutase [Cu-Zn]	SOD3	−3.74
		Q92781	11-cis retinol dehydrogenase	RDH5	−6.65
*Glutathione transferase activity* (GO:0004364)	0.00	P09211	Glutathione S-transferase P	GSTP1	−2.35
		P28161	Glutathione S-transferase Mu 2	GSTM2	−2.55
		P30711	Glutathione S-transferase theta-1	GSTT1	−3.49
		P09488	Glutathione S-transferase Mu 1	GSTM1	−5.63

Functional categories were retrieved from the DAVID database. The *p* value for each term is shown. Relative abundance change of proteins is indicated with the corresponding Zq value for each comparison. These values can also be found in the extended Supplementary Table S3, where a colour scale in red and blue colours is represented for up- and down-regulated proteins, respectively.
