# Supplementary material for: Proteome-wide alterations on adipose tissue from obese patients as age-, diabetes- and gender-specific hallmarks
Source: Sci Rep. 2016 May 10;6:25756. doi: 10.1038/srep25756 (PMC4861930; doi:10.1038/srep25756)
Supplement: Supplementary Information [file srep25756-s1.pdf]

Proteome-wide alterations on adipose tissue from obese patients as age-, diabetes- and gender-specific hallmarks

María Gómez-Serrano[1†], Emilio Camafeita[2†], Eva García-Santos[1], Juan A. López[2], Miguel A. Rubio[3], Andrés Sánchez-Pernaute[4], Antonio Torres[4], Jesús Vázquez[2], Belén Peral[1,5]*

[1] Instituto de Investigaciones Biomédicas, Alberto Sols, (IIBM); Consejo Superior de Investigaciones Científicas & Universidad Autónoma de Madrid (CSIC-UAM); Madrid, 28029; SPAIN.

[2] Laboratory of Cardiovascular Proteomics, Centro Nacional de Investigaciones Cardiovasculares (CNIC); Madrid, 28029; SPAIN.

[3] Department of Endocrinology, Hospital Clínico San Carlos (IDISSC), Facultad de Medicina, Universidad Complutense; Madrid, 28040; SPAIN

[4] Department of Surgery, Hospital Clínico San Carlos (IDISSC), Facultad de Medicina, Universidad Complutense; Madrid, 28040; SPAIN.

[5] CIBER de Fisiopatología de la Obesidad y Nutrición (CIBEROBN), Instituto de Salud Carlos III (ISCIII); SPAIN.

[†] These authors contributed equally to this work.

* Corresponding Author.

SUPPLEMENTARY INFORMATION

- **Supplementary Figures and Legends**
- **Supplementary Tables and Headings**
- **Supplementary Methods**
- **References**

SUPPLEMENTARY FIGURES AND LEGENDS

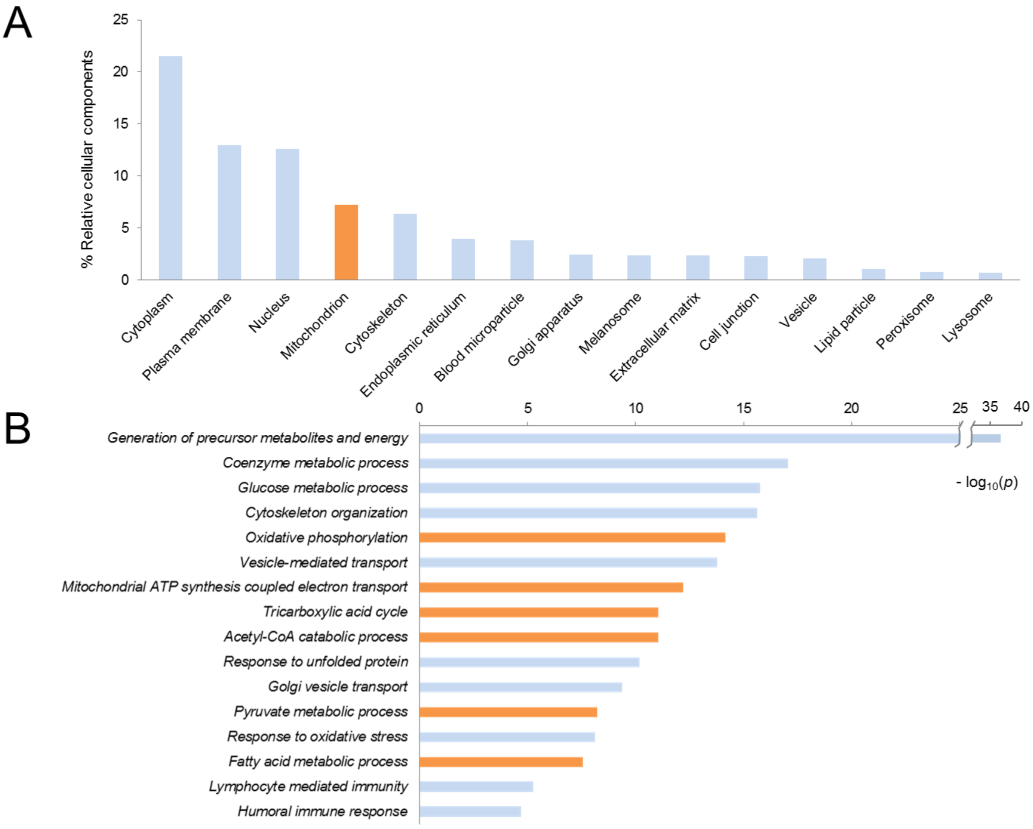

Supplementary Figure S1. Function of the VAT proteome from obese patients. (A) Relative proportion of the most representative GOCC terms found in the proteins identified. **(B)** Enrichment analysis of GOBP terms. The bars represent – $\log_{10}(p)$, where p is the enrichment p value. Representative GOBPs are shown. Mitochondrion-associated GOBPs are highlighted in orange. See also Supplementary Table S2B.

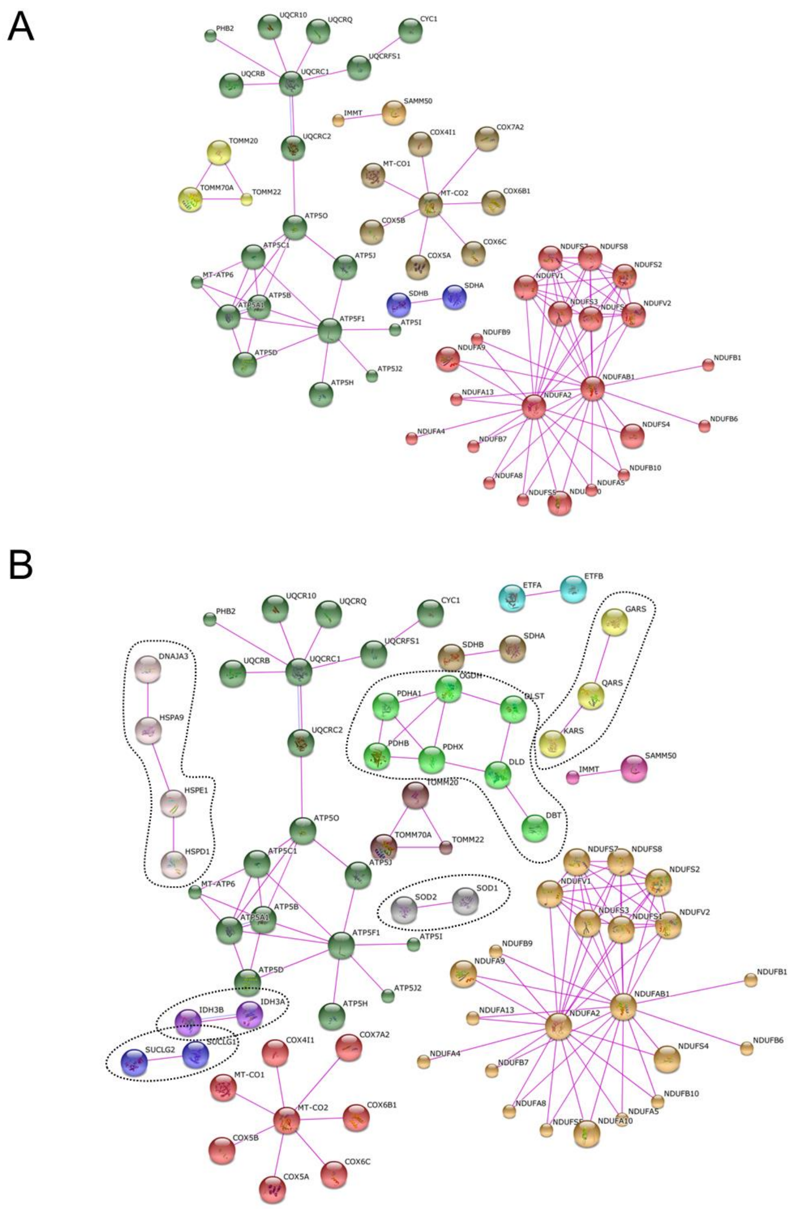

Supplementary Figure S2. Network analysis of mitochondrial proteins down-regulated in the age and T2DM comparisons. (A) Interactome of proteins belonging to the *mitochondria* cluster in the age comparison (n=158). **(B)** Interactome of proteins belonging to the *mitochondria* cluster in the T2DM comparison (n=214). Only experimentally validated protein-protein interactions from the STRING database [1] were considered with a 0.95 confidence score threshold. Different interacting node clusters are indicated with colours. Exclusive interacting node clusters in T2DM *mitochondria* cluster are rounded with a dashed line.

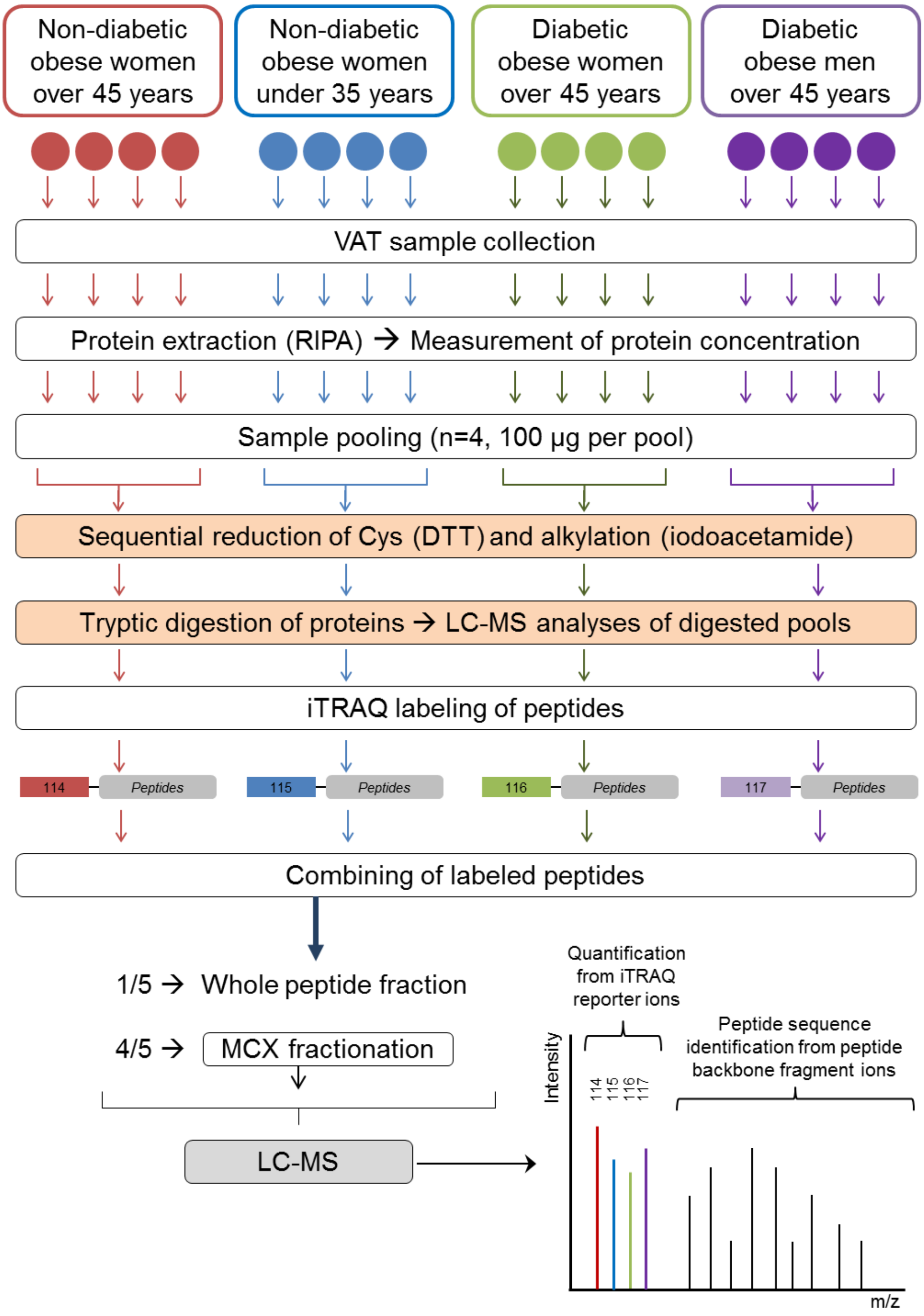

Figure S3. Proteomics procedures. The protein extracts obtained from each patient sample (n = 16) were quantitated using two different methods (in duplicate) to ensure proper sample pooling. To minimize differences among groups, reduction of Cys (with DTT) and on-filter tryptic protein digestion (orange boxes) were carried out with protein pools. After tryptic

digestion, a small amount of each peptide pool (~ 2 µg) was analyzed by LC-MS to check for comparable digestion across groups. Thereafter the peptide pools were tagged with their corresponding iTRAQ labels and equally mixed. The so-obtained mixture of differentially labeled peptides was split into an unfractionated peptide sample (one-fifth) and eight peptide samples obtained by MCX fractionation (four-fifths). LC-MS analyses of these nine samples allowed the identification of peptide sequences based on peptide backbone fragment ions and the quantification of their relative abundance across sample groups based on the intensity of iTRAQ reporter ions showing in the low m/z region. A detailed description of experimental procedures is provided in Supplementary Methods.

SUPPLEMENTARY TABLES

Supplementary Table S1. Clinical characteristics of patients enrolled in proteome profiling. Clinical variables are expressed as mean ± SD. One-way ANOVA (using Bonferroni's *post hoc* test) was used to compare clinical variables. Significance was set at $p < 0.05$.

	Non-diabetic obese women over 45 years (n=4)	*Non-diabetic obese women under 35 years* (n=4)	*Diabetic obese women over 45 years* (n=4)	*Diabetic obese men over 45 years* (n=4)	*p*
Age (years)	51 ± 5 *	31.75 ± 4	50 ± 6 *	49.75 ± 3 *	0.00
Body Mass Index, BMI (kg/m^2)	44.75 ± 6.85	43.65 ± 3.37	46.63 ± 6.05	44.18 ± 5.86	0.89
Waist circumference (cm)	120.50 ± 8.23	117.25 ± 8.77	127.25 + 2.99	128.25 ± 14.22	0.33
Fasting glucose (mmol/L)	5.64 ± 0.63	5.29 ± 0.27	8.78 ± 4.12	9.68 ± 3.89	0.12
HbA1c (%)	5.60 ± 0.14	5.55 ± 0.24	7.50 ± 2.00	7.03 ± 1.43	0.10
Fasting triglycerides (mmol/L)	1.68 ± 0.53	1.33 ± 0.32	2.11 ± 1.19	1.61 ± 0.65	0.55
Total cholesterol (mmol/L)	5.28 ± 0.52	4.95 ± 0.67	4.46 ± 1.46	4.29 ± 1.49	0.60
cLDL (mmol/L)	3.11 ± 0.43	2.91 ± 0.30	2.31 ± 1.01	2.38 ± 1.15	0.46
cHDL (mmol/L)	1.40 ± 0.15	1.44 ± 0.38	1.20 ± 0.14	1.16 ± 0.22	0.29

* $p < 0.001$, significant differences in comparison to *non-diabetic obese women under 35 years,* by Bonferroni's *post hoc* test.

SUPPLEMENTARY TABLES HEADINGS (Excel Dataset Files)

Supplementary Table S2. Characterization of VAT proteome in obese patients.

Table S2A. Proteins identified in VAT samples at 1% FDR. UniProt accession codes, Fasta protein description and the corresponding gene names (N/A means "Not Assigned") are displayed. Unique peptides and spectral counts are also reported. The FDR for peptide identification was set at 1%.

Table S2B. GO enrichment analysis of the 2,525 VAT proteins identified. 'Count' represents the number of proteins within the corresponding categories. The percentage represents the proportion of gene-coded proteins in the corresponding GOBP. The magnitude of GOPB enrichment is measured by the corresponding fold enrichment, and *p* values indicate the significance level. Only GOBP terms with $p < 0.05$ are shown. Green cells correspond to GOBPs represented in Supplementary Fig. S1B.

Supplementary Table S3. VAT proteins quantitated with the WSPP model. X'q is calculated as a weighted average of the peptides used to quantify the protein corrected by the grand mean (calculated as a weighted average of the total protein values). **Wq** refers to the statistical weight associated with the protein, calculated from the corresponding peptide weights and the protein variance (the statistical weights are the inverses of variances). **Zq** corresponds to the standardized variable, which is defined at the protein level as the mean-corrected $\log_2$ratio expressed in units of standard deviation. **FDRq** refers to the False Discovery Rate at the protein level. **(1)** Indicates 114 *vs*. 115 or age comparison, **(2)** indicates 116 *vs*. 114 or T2DM comparison, and **(3)** indicates 116 *vs*. 117 or gender comparison.

Supplementary Table S4. Enrichment analysis of non-overlapping DAPs in the age comparison using DAVID software. 'Count' represents the number of proteins within the corresponding category. The percentage represents the proportion of gene-coded proteins in this category. The magnitude of category enrichment is measured by the corresponding fold enrichment, and *p* values indicate the significance level. Only terms with $p < 0.05$ are shown.

Supplementary Table S5. Enrichment analysis of non-overlapping DAPs in the T2DM comparison using DAVID software. 'Count' represents the number of proteins within the corresponding category. The percentage represents the proportion of gene-coded proteins in this category. The magnitude of category enrichment is measured by the corresponding fold enrichment, and *p* values indicate the significance level. Only terms with $p < 0.05$ are shown.

Supplementary Table S6. Enrichment analysis of non-overlapping DAPs in the gender comparison using DAVID software. 'Count' represents the number of proteins within the corresponding category. The percentage represents the proportion of gene-coded proteins in this category. The magnitude of category enrichment is measured by the corresponding fold enrichment, and *p* values indicate the significance level. Only terms with $p < 0.05$ are shown.

Supplementary Table S7. Clustering analysis in the age comparison. Clustering of 62 differentially represented functional categories ($n \geq 5$, FDR < 0.05) resulted in eight clusters containing a number of categories made up of similar proteins. The number of proteins present in each category is indicated by "n". Grey cells indicate the most representative categories within the cluster shown in Fig. 4D.

Supplementary Table S8. Clustering analysis in the T2DM comparison. Clustering of 64 differentially represented functional categories ($n \geq 5$, FDR < 0.05) resulted in five clusters containing a number of categories made up of similar proteins. The number of proteins present in each category is indicated by "n". Grey cells indicate the most representative categories within the cluster shown in Fig. 4E.

Supplementary Table S9. Clustering analysis in the gender comparison. Clustering of 46 differentially represented functional categories ($n \geq 5$, FDR < 0.05) resulted in eight clusters containing a number of categories made up of similar proteins. The number of proteins present in each category is indicated by "n". Grey cells indicate the most representative categories within the cluster shown in Fig. 4F.

Supplementary Table S10. Mitochondrial proteins related to the onset of T2DM according to TPMS. Mitochondrial proteins (according to the UniProt database annotations) presenting differential activation or inhibition functional states ($p < 0.05$) are shown.

**Comparison**	**UniProt**	**Protein name**	**Symbol**	**Differential state**	**Group of patients**	***p* value**
**AGE**	P12931	Proto-oncogene tyrosine-protein kinase Src	SRC	Activation	**Younger** non-diabetic women	0.029
	P29353	SHC-transforming protein 1	SHC1	Inhibition	**Older** non-diabetic women	0.000
	Q13114	TNF receptor-associated factor 3	TRAF3	Inhibition	**Older** non-diabetic women	0.024
**T2DM**	P12931	Proto-oncogene tyrosine-protein kinase Src	SRC	Activation	Older **non-diabetic** women	0.011
	P29353	SHC-transforming protein 1	SHC1	Activation	Older **non-diabetic** women	0.028
	P29353	SHC-transforming protein 1	SHC1	Inhibition	Older **diabetic** women	0.043
	P53779	Mitogen-activated protein kinase 10	MAPK10	Inhibition	Older **non-diabetic** women	0.003

SUPPLEMENTARY METHODS

Study design

The main aim of this study was to analyze the visceral adipose tissue (VAT) proteome from age-, T2DM- and gender-matched obese patients using high-throughput proteomics and systems biology methods to investigate the influence of T2DM, aging and gender in obesity in the same experiment. The study design is outlined in Fig. 1. VAT samples were collected from a subgroup of 16 obese patients (BMI $\geq$ 40 kg/m^2) with highly homogenous clinical features selected out of 50 subjects initially enrolled. All the patients were of Caucasian origin submitted to bariatric surgery. For proteome profiling, the 16 patients were split in four groups: non-diabetic women over 45 years, non-diabetic women under 35 years, diabetic women over 45 years and diabetic men over 45 years. Inclusion and exclusion criteria are outlined below. The experimental proteomic procedures, based on peptide iTRAQ labeling and LC-MS analysis, are depicted in Fig. S3. Briefly, protein extracts obtained from whole fat biopsies were equally pooled and digested following a filter-aided sample preparation (FASP) protocol. The so-obtained peptides were tagged with iTRAQ labels and analyzed by LC-MS. Peptide identification was achieved through the SEQUEST algorithm implemented in Protein Discoverer 1.4, and protein quantification was based on the WSPP statistical model. At 1% FDR, 2,329 gene-coded proteins were identified and about 200-250 proteins revealed differentially abundant in each of the three comparisons performed. Category enrichment analyses using DAVID software together with functional category clustering analyses were also achieved. Results validation was performed by Western Blot and immunohistochemistry methods with an additional set of subjects. These experiments were blinded. Finally to gain an integrated comprehensive view of the VAT proteome dynamics under T2DM and aging conditions, systems biology analyses based on artificial intelligence and pattern recognition techniques were also performed.

Biological samples

VAT was obtained from 16 obese patients (BMI $\geq$ 40 kg/m^2) selected out of 50 subjects initially enrolled who underwent bariatric surgery. The biopsies were obtained after an overnight fast, washed three times in phosphate-buffered saline (PBS), partitioned into pieces, and immediately frozen in liquid nitrogen and stored at −80 °C until protein extraction. The surgeon aimed to obtain the samples at the beginning of the surgery and from the same anatomical location around the *omemtum* in all subjects. All patients reported that their body weight had been stable for at least three months before the study. None of the non-diabetic subjects suffered T2DM or other obesity associated co-morbidity (hypertension, dyslipidemia, obstructive sleep apnea syndrome or CVD), and none of them was being treated. Inclusion criterion for the diabetic group was suffering for T2DM for at least two years. T2DM was defined by fasting plasma glucose $\geq$ 7 mmol/L and HbA1c $\geq$ 6.5 %). All T2DM subjects were being treated with oral anti-diabetic drugs and in two cases with insulin in order to control the co-morbidities. The clinical characteristics of the patients enrolled are described in Table S1. **Exclusion criteria** for all patients included in this study were: i) clinically significant hepatic, neurological, or other major systemic disease, including malignancy; ii) history of drug or alcohol abuse, defined as > 80 g/day, or serum transaminase activity more than twice the upper normal range limit; iii) elevated serum creatinine concentrations; iv) acute major cardiovascular event in the previous 6 months; v) acute illnesses and current evidence of chronic inflammatory or infectious diseases; and vi) mental illness rendering the subjects unable to understand the nature, scope, and possible consequences of the analysis.

Preparation of protein extracts

Proteins were extracted from VAT (about 100 mg) in radioimmunoprecipitation assay buffer (RIPA) (25 mM Tris-HCl pH 7.6, 150 mM NaCl, 1% NP-40, 1% sodium deoxycholate, 0.1% SDS) supplemented with a protease inhibitor cocktail (*Halt Protease Inhibitor Cocktail*, Thermo Scientific) [2] using the *Sample Grinding Kit* (GE Healthcare) according to the manufacturer's instructions. For a more precise sample pooling, two different methods were

used to measure the concentration of protein extracts: *Pierce* BCA Protein Assay (Thermo Scientific) and *Direct Detect* infrared-based quantification system (EMD Millipore). The assays were performed in duplicate by both methods with less than 1% variation between measurements. A protein pool was prepared with proteins from the four patients included in each group.

On-filter digestion

For each pool, a total of 100 µg of proteins (n=4, 25 µg per sample) were digested on-filter (FASP, *Protein Digestion Kit*, Expedeon) [3] following the manufacturer's instructions. First, proteins were boiled in the presence of 50 mM dithiothreitol (DTT). Then the protein pools were loaded onto the filter and washed in urea solution by repeated centrifugation steps. Thiols were carboxyamidomethylated with iodoacetamide (IAA) and excess reagent was removed by repeated washing with urea solution and 50 mM ammonium bicarbonate by ultrafiltration steps. Finally, protein extracts were digested by overnight incubation at 37 °C with 30 ng/µL trypsin at a 40:1 protein:trypsin (w/w) ratio in 50 mM ammonium bicarbonate solution. The filtrate containing the digested protein fraction was obtained after filter extraction with 500 mM sodium chloride by ultracentrifugation at 10,000 rpm for 10 min. The resulting peptides were desalted onto OASIS HLB C18 cartridges (Waters) and vacuum-dried. The digestion yield in the so-obtained peptide pools was checked by LC-MS analysis of a 2 µg aliquot of each pool.

iTRAQ labeling

Protein pooled samples were labeled as summarized in Figure 1 and S3. For iTRAQ labeling, the dried peptides were taken up in 30 µL of 0.5 M triethylammoniumbicarbonate (TEAB) buffer and labeled with 70 µL of the corresponding iTRAQ reagent in 70% (v/v) ethanol for 1 h at room temperature. After this, 100 µL of 0.5% (v/v) trifluoroacetic acid (TFA) was added and samples were incubated 15 min to completely stop the labeling reaction. The peptide samples were mixed, vacuum concentrated and diluted in 200 µL of 1% (v/v) TFA for desalting on Oasis

HLB C18 cartridges (Waters). One-fifth of the tagged peptides were directly analyzed by LC-MS, and the remaining four-fifths were subject to MCX fractionation.

MCX peptide fractionation

The iTRAQ-labeled peptides were suspended in 5 mM ammonium formate (AF) with 25% (v/v) acetonitrile (ACN), pH 3.0, and separated into 8 fractions using MCX Oasis cartridges (Waters) with the following elution buffers: 0.5, 1.0 and 1.5 M AF, pH 3.0, containing 25% (v/v) ACN; 0.5, 1.25 M FA, pH 3.0, containing 37% (v/v) ACN and 1M AF, pH 3.0, containing 50% (v/v) ACN. Peptide fractions were desalted using MicroSpin Columns C18 (The Next Group), vacuum-dried and kept at 4 ºC for later LC-MS analysis. Fractions 1&8 and 2&7 were pooled for LC-MS analysis.

LC-MS analyses and protein identification

High-resolution analysis of iTRAQ-labeled peptides was carried out on an Easy nLC 1000 nano-HPLC apparatus (Thermo Scientific) coupled to a hybrid quadrupole-orbitrap mass spectrometer (Q Exactive, Thermo Scientific). Peptides were suspended in 0.1% formic acid and then loaded onto an PepMap100 C18 LC pre-column (75 µm I.D., 2 cm, Thermo Scientific) and eluted on line onto an analytical NanoViper PepMap™ 100 C18 LC column (75 µm I.D., 50 cm, Thermo Scientific) with a continuous gradient consisting of 8-31% B for 240 min and 31-90% B for 2 min (B = 90% acetonitrile, 0.1% formic acid) at 200 nL/min. Peptides were ionized using a Picotip emitter nanospray needle (New Objective). Each MS run consisted of enhanced FT-resolution spectra (120,000 resolution) in the 390–1,200 m/z range followed by data-dependent MS/MS spectra of the 20 most intense parent ions acquired along the chromatographic run. The AGC target value for the survey scan was set to 10^6. Fragmentation in the Orbitrap was performed at 33% normalized collision energy with a target value of 10,000 ions. The full target was set to 30,000, with 1 microscan and 100 ms injection time, and the dynamic exclusion was set to 0.5 min. A total of 7 MS data sets, one from the unfractionated material and six from the corresponding MCX fractions were registered with 28 h total

acquisition time. For peptide identification the MS/MS spectra were searched with the SEQUEST algorithm implemented in Proteome Discoverer 1.4.0.29 (Thermo Scientific). Database searching against human protein sequences from the Uniprot database (September 2014, 147,615 entries) was performed with the following parameters: trypsin digestion with 2 maximum missed cleavage sites; precursor and fragment mass tolerances of 2 Da and 0.02 Da, respectively; carbamidomethyl cysteine as fixed modification and methionine oxidation as a dynamic modification. For iTRAQ labeled peptides, N-terminal and Lys iTRAQ modifications were selected as fixed modifications. The results were analyzed using the probability ratio method [4] with an additional filtering for a precursor mass tolerance of 10 ppm and the FDR for peptide identification was calculated based on the search results against a decoy database using the refined method [5].

Statistical analyses

Descriptive results of continuous variables are expressed as mean ± standard deviation (SD). Statistical analyses were performed using the Statistical Package for Social Science software (v. 22, SPSS, Inc). One-way ANOVA (using Bonferroni's *post hoc* test) was used to compare the anthropometrical and clinical data from the patients, and the statistical significance was set at $p < 0.05$. Peptide and protein abundance changes were assessed at 1% peptide FDR using the iTRAQ reporter ion intensities retrieved from MS/MS scans by QuiXoT software [6,7] as inputs to the WSPP model [8]. This model circumvents the statistical issues reported for protein quantitation from iTRAQ-labeled peptides [9] and, by decomposing the different variance components, takes into account variance non-homogeneity and the highly unbalanced structure of this kind of data [8]. The model also provides a robust framework for the full integration of quantitative and error information from a given level to a superior level (*e.g.* peptide to protein). The threshold for differential protein abundance was at $|Zq| \geq 2$. In addition, *ca.* $2.3 \cdot 10^6$ functional category terms with relevance to adipose tissue and obesity were retrieved from the DAVID database [10,11] and used with the WSPP model to determine statistically significant

changes at the protein function level as previously described [12-14]. An averaged $\log_2$ ratio, X'c, was calculated for each functional category as a weighted average of the proteins that belong to the category. Categories with at least five protein components and FDR < 0.05 were considered as significantly differential and were subjected to cluster analysis.

Therapeutic Performance Mapping System (TPMS) analyses

Based on artificial intelligence and pattern recognition techniques [15,16] *Therapeutic Performance Mapping System* (TPMS, Anaxomics Biotech.) creates mathematical models that integrate all the available biological, pharmacological and medical knowledge to simulate human physiology *in silico* [17-22]. The process encompasses four steps: 1) Collection of scientific knowledge based on hand-curated databases that relate biological processes to their molecular effectors (including a specific TD2M characterization); 2) Preparation of a human biological network focused in the area around the disease of interest (obesity and T2DM) based on data retrieved from both public and private external databases such as KEGG [23], BioGRID [24], IntAct [25], REACTOME [26] and MINT [27]; 3) Mathematical model generation, whereby the biological map is transformed into a mathematical model capable of both reproducing existing knowledge and predicting new data (which is achieved by training the biological network with the *Truth Table*, a collection of mathematical restrictions that include the available biological knowledge about the constructed networks, together with knowledge derived from DrugBank [28], and the differentially abundant proteins (DAPs) in the present iTRAQ experiment); and 4) Extraction of biological and clinical conclusions. To extract conclusions, each mathematical model is challenged with the stimulus (in our case the most relevant proteins triggering T2DM, defined as the proteins most connected to other T2DM-related proteins, according to the molecular characterization of the disease based on the scientific knowledge to date) and a response (in our case effectors of T2DM). Sampling methods were used to describe with high capability all plausible relationships between the stimulus and the response. As the number of restrictions is always smaller than the number of parameters required by the algorithm, any process modeled by TPMS considers a population of

different solutions, currently set around 10^6-10^9 since this interval is estimated to faithfully portray nature. Then, TPMS traces the most probable pathways (in biological and mathematical terms) among all the possible pathways leading from the stimulus to the response through the biological network (step 4). In other words, it identifies the most probable molecular mechanisms that achieve a physiological response, here, T2DM. Once the mathematical models are generated, the level of functional modulation of each protein in the models is compared between them, contrasting older *vs*. younger women (age comparison) and diabetic *vs*. non-diabetic women (T2DM comparison). The times that a protein appears as activated or inhibited in the mechanisms of action that configure the mathematical solutions to the models are counted and compared, and statistically differential proteins (p value < 0.05) between each pair of compared models are considered. Systems biology results indicating activation/inhibition functional states have to be comprehended as the most probable state considering mechanisms leading to T2DM. Consequently, mechanisms significantly over-represented in non-diabetic patients have to be recognized as "mechanisms that must be affected" to reach T2DM according to the publicly available biological knowledge. On the contrary, mechanisms over-represented in T2DM patients may reflect the maintenance of diabetic condition by itself. An overview of the methodology performed in this study is shown in Fig. 6A.

REFERENCES

1 Szklarczyk, D. *et al.* STRING v10: protein-protein interaction networks, integrated over the tree of life. *Nucleic Acids Res* **43**, D447-452, doi:10.1093/nar/gku1003 (2015).

2 Perez-Perez, R. *et al.* Attenuated metabolism is a hallmark of obesity as revealed by comparative proteomic analysis of human omental adipose tissue. *J Proteomics* **75**, 783-795, doi:10.1016/j.jprot.2011.09.016 (2012).

3 Wieckowski, M. R., Giorgi, C., Lebiedzinska, M., Duszynski, J. & Pinton, P. Isolation of mitochondria-associated membranes and mitochondria from animal tissues and cells. *Nat Protoc* **4**, 1582-1590, doi:10.1038/nprot.2009.151 (2009).

4 Martinez-Bartolome, S. *et al.* Properties of average score distributions of SEQUEST: the probability ratio method. *Mol Cell Proteomics* **7**, 1135-1145, doi:10.1074/mcp.M700239-MCP200 (2008).

5 Navarro, P. & Vazquez, J. A refined method to calculate false discovery rates for peptide identification using decoy databases. *J Proteome Res* **8**, 1792-1796, doi:10.1021/pr800362h (2009).

6 Lopez-Ferrer, D., Ramos-Fernandez, A., Martinez-Bartolome, S., Garcia-Ruiz, P. & Vazquez, J. Quantitative proteomics using 16O/18O labeling and linear ion trap mass spectrometry. *Proteomics* **6 Suppl 1**, S4-11, doi:10.1002/pmic.200500375 (2006).

7 Ramos-Fernandez, A., Lopez-Ferrer, D. & Vazquez, J. Improved method for differential expression proteomics using trypsin-catalyzed 18O labeling with a correction for labeling efficiency. *Mol Cell Proteomics* **6**, 1274-1286, doi:10.1074/mcp.T600029-MCP200 (2007).

8 Navarro, P. *et al.* General statistical framework for quantitative proteomics by stable isotope labeling. *J Proteome Res* **13**, 1234-1247, doi:10.1021/pr4006958 (2014).

9 Luo, R. & Zhao, H. Protein quantitation using iTRAQ: Review on the sources of variations and analysis of nonrandom missingness. *Stat Interface* **5**, 99-107 (2012).

10 Huang da, W., Sherman, B. T. & Lempicki, R. A. Systematic and integrative analysis of large gene lists using DAVID bioinformatics resources. *Nat Protoc* **4**, 44-57, doi:10.1038/nprot.2008.211 (2009).

11 Huang da, W., Sherman, B. T. & Lempicki, R. A. Bioinformatics enrichment tools: paths toward the comprehensive functional analysis of large gene lists. *Nucleic Acids Res* **37**, 1-13, doi:10.1093/nar/gkn923 (2009).

12 Jorge, I. *et al.* The human HDL proteome displays high inter-individual variability and is altered dynamically in response to angioplasty-induced atheroma plaque rupture. *J Proteomics* **106**, 61-73, doi:10.1016/j.jprot.2014.04.010 (2014).

13 Martin-Alonso, M. *et al.* Deficiency of MMP17/MT4-MMP proteolytic activity predisposes to aortic aneurysm in mice. *Circ Res* **117**, e13-26, doi:10.1161/CIRCRESAHA.117.305108 (2015).

14 Quiros, P. M. *et al.* ATP-dependent Lon protease controls tumor bioenergetics by reprogramming mitochondrial activity. *Cell Rep* **8**, 542-556, doi:10.1016/j.celrep.2014.06.018 (2014).

15 Goldberg, D. E. *Genetic Algorithms in Search, Optimization and Machine Learning*. (Addison-Wesley Longman Publishing Co., Inc., 1989).

16 Kuncheva, L. I. *Combining Pattern Classifiers: Methods and Algorithms*. (Wiley-Interscience, 2004).

17 Coma, M. *et al.* Combination therapies for treating neurological disorders. (Google Patents, 2013).

18 Badiola, N. *et al.* The proton-pump inhibitor lansoprazole enhances amyloid beta production. *PLoS One* **8**, e58837, doi:10.1371/journal.pone.0058837 (2013).

19 Kotelnikova, E. *et al.* Signaling networks in MS: a systems-based approach to developing new pharmacological therapies. *Mult Scler* **21**, 138-146, doi:10.1177/1352458514543339 (2015).

20 Coma, M. *et al.* Methods for diagnosing perceived age on the basis of an ensemble of phenotypic features. *Clin Cosmet Investig Dermatol* **7**, 133-137, doi:10.2147/CCID.S52257 (2014).

21 Pujol, A., Mosca, R., Farres, J. & Aloy, P. Unveiling the role of network and systems biology in drug discovery. *Trends Pharmacol Sci* **31**, 115-123, doi:10.1016/j.tips.2009.11.006 (2010).

22 Aloy, P. & Russell, R. Targeting and tinkering with interaction networks. *FEBS Lett* **582**, 1219, doi:10.1016/j.febslet.2008.02.069 (2008).

23 Kanehisa, M. *et al.* From genomics to chemical genomics: new developments in KEGG. *Nucleic Acids Res* **34**, D354-357, doi:10.1093/nar/gkj102 (2006).

24 Salwinski, L. *et al.* Recurated protein interaction datasets. *Nat Methods* **6**, 860-861, doi:10.1038/nmeth1209-860 (2009).

25 Kerrien, S. *et al.* The IntAct molecular interaction database in 2012. *Nucleic Acids Res* **40**, D841-846, doi:10.1093/nar/gkr1088 (2012).

26 Croft, D. *et al.* The Reactome pathway knowledgebase. *Nucleic Acids Res* **42**, D472-477, doi:10.1093/nar/gkt1102 (2014).

27 Licata, L. *et al.* MINT, the molecular interaction database: 2012 update. *Nucleic Acids Res* **40**, D857-861, doi:10.1093/nar/gkr930 (2012).

28 Wishart, D. S. *et al.* DrugBank: a knowledgebase for drugs, drug actions and drug targets. *Nucleic Acids Res* **36**, D901-906, doi:10.1093/nar/gkm958 (2008).
